# Diethyl butylmalonate attenuates cognitive deficits and depression in 5×FAD mice

**DOI:** 10.3389/fnins.2024.1480000

**Published:** 2024-11-11

**Authors:** Lai Yuan, Ge Song, Wangwei Xu, Shuni Liu, Yongsheng Zhang, Wei Pan, Xiaohui Ding, Linlin Fu, Qisi Lin, Fenfen Sun

**Affiliations:** ^1^Jiangsu Key Laboratory of Immunity and Metabolism, Department of Pathogen Biology and Immunology, Xuzhou Medical University, Xuzhou, China; ^2^The First Clinical Medical College, Xuzhou Medical University, Xuzhou, China; ^3^Jiangsu Key Laboratory of New Drug Research and Clinical Pharmacy, Xuzhou Medical University, Xuzhou, China; ^4^Suqian Affiliated Hospital of Xuzhou Medical University, Suqian, China

**Keywords:** Alzheimer’s disease, cognitive, depression, neuroinflammation, synaptic plasticity, diethyl butylmalonate

## Abstract

**Background:**

Alzheimer’s disease (AD), characterized by cognitive impairment and depression, is currently one of the intractable problems due to the insufficiency of intervention strategies. Diethyl butylmalonate (DBM) has recently attracted extensive interest due to its anti-inflammatory role in macrophages. However, it is still unknown whether DBM has a beneficial effect on cognitive deficits and depression.

**Methods:**

DBM was administrated to 5×FAD and C57BL/6J mice by intraperitoneal injection. Novel object recognition, Y-maze spatial memory, Morris water maze and nest building tests were used to evaluate cognitive function. Moreover, the tail suspension test, forced swimming test, open field test and the elevated plus maze test were used to assess depression. Transmission electron microscopy, Golgi-Cox staining, immunofluorescence, RT-qPCR and western blot were utilized to determine the neuropathological changes in the hippocampus and amygdala of mice.

**Results:**

Multiple behavioral tests showed that DBM effectively mitigated cognitive deficit and depression in 5×FAD mice. Moreover, DBM significantly attenuated synaptic ultrastructure and neurite impairment in the hippocampus of 5×FAD mice, paralleled by the improvement of the deficits of PSD95 and BDNF proteins. In addition, DBM decreased the accumulation of microglia and downregulated neuroinflammation in the hippocampus and amygdala of 5×FAD mice.

**Conclusion:**

This study provides evidence that DBM ameliorates cognitive deficits and depression via improvement of the impairment of synaptic ultrastructure and neuroinflammation, suggesting that DBM is a potential drug candidate for treating AD-related neurodegeneration.

## 1 Introduction

Alzheimer’s disease (AD) is a multifactorial neurodegenerative disorder and the leading cause of dementia (Paul, 2022). AD patients are characterized by cognitive impairment, including memory deficits and depression (Lyketsos et al., 2011; Kogan et al., 2000; Koenig et al., 2016). Neuroinflammation is critical in AD progression via impairing synaptic plasticity and neurite outgrowth (Hou et al., 2021; Hartmann et al., 2024; Bohlson and Tenner, 2023; Li et al., 2023). Neuroinflammation plays a vital role in the AD progression (Leng and Edison, 2021). In AD, activated microglia release a range of proinflammatory cytokines, including IL-1β, IL-6, and TNF-α (Cai et al., 2019), directly inducing neuronal apoptosis and synaptic dysfunction (Zhao et al., 2019). These activated microglia can also engulf synapses, compromising synaptic structure (Hong et al., 2016). Consequently, reducing neuroinflammation may help alleviate cognitive impairment by enhancing synaptic plasticity and preserving synaptic ultrastructure in AD.

Succinate dehydrogenase (SDH), an enzyme in Kreb’s cycle, is a arbiter of pro-inflammatory response in macrophages (Mills et al., 2016). It is reported that SDH governs the increased inflammatory gene expression and reciprocally decreased anti-inflammatory gene expression via succinate oxidation (Mills et al., 2016). Diethyl butylmalonate (DBM) is known to be a competitive inhibitor of SDH. It rapidly hydrolyzes to form malonate, preventing succinate accumulation and oxidation, thereby exerting an anti-inflammatory effect by inhibiting ROS production (Zhang et al., 2021; Fan et al., 2022; Prag et al., 2022). It is reported that DBM administration can alleviate brain damage in a mouse model of cardiac arrest (Leng and Edison, 2021). Additionally, recent research has demonstrated that DBM suppresses microglial activation and reduces neuroinflammation in LPS-stimulated microglia (Sangineto et al., 2023). However, it remains largely enigmatic whether DBM could improve cognitive impairment and depression in AD.

Transgenic mice have become essential tools for exploring the neuropathology of AD and evaluating potential therapeutic agents (Wong et al., 2002). Here, utilizing the 5×FAD mice model, which showed cognitive impairment and depression (Jawhar et al., 2012; Xu et al., 2018), we comprehensively evaluated the protective effects of DBM on the condition of AD. Our results indicate that DBM treatment improves cognitive function and alleviates depression in these transgenic mice, alongside enhancements in synaptic ultrastructure, neurite growth, and reductions in neuroinflammation. This suggests that DBM holds promise as a therapeutic molecule for preventing neurodegenerative diseases, including AD.

## 2 Materials and methods

### 2.1 Animals

C57BL/6J mice were obtained from Jiangsu Jicui Pharmaceutical Technology Corporation (Jiangsu Province, China) and the transgenic mouse line 5×FAD (MMRRC catalog #034840-JAX, RRID: MMRRC_034840-JAX) were purchased from the Jackson Laboratory. 5×FAD mice overexpress the 695-amino acid isoform of the human amyloid precursor protein (APP695) carrying the Swedish, London, and Florida mutations under the control of the murine Thy-1 promoter. They were housed in environmentally controlled conditions (temperature 24°C, 12 h light/dark cycle) and given free access to standard food and water in specific pathogen free (SPF) experimental animal Center of Xuzhou Medical University. The mice were acclimatized for 1 week before the experiment. All animal care and experiments were carried out in strict accordance with the recommendation of the Guide for the Care and Use of Laboratory Animals of the Ministry of Health (China) and approved by the Ethics Committee of Xuzhou Medical University (Xuzhou, China, SCXK (Su) 2020-0048).

### 2.2 Experimental design

5×FAD mice (4 month old) and C57BL/6J mice (4 month old) were randomly divided into the following four groups (12 mice for each group, including 6 males and 6 females): (I) C57BL/6J mice received phosphate buffer solution (PBS) as vehicle control (Con + Veh) group; (II) C57BL/6J mice received intraperitoneal injections (two times weekly) of 40 mg/kg DBM (Cat. 112038-100ML, Sigma-Aldrich, St. Louis, United States) as Con + DBM group; (III) 5×FAD group as AD model for mice received PBS as 5×FAD + Veh group; (IV) 5×FAD + DBM group received DBM (two times weekly) with intraperitoneal injections in 5×FAD mice. Following 4 weeks of intervention, a series of behavioral tests were performed. Mice were then anesthetized with pentobarbitone sodium (100 mg/kg body weight) and sacrificed 3 days after behavioral tests. The fresh hippocampus and amygdala tissues were collected and preserved at −80°C for western blotting and RT-qPCR. The whole brains were taken and placed in Golgi fixative for Golgi-Cox staining. For transmission electron microscope, the left hippocampus was sectioned and cut into 1 cm^3^ pieces and stored in electron microscope fixative. For immunofluorescence analysis, the whole brains were placed in a 4% paraformaldehyde-fixed solution and then transferred into a 30% sucrose solution for dehydration and cryoprotection.

### 2.3 Behavioral tests

To investigate the impact of DBM administration on spontaneous rodent behaviors and recognition memory and depression in 5×FAD mice, behavioral tests were conducted, including Morris water maze, Y-maze spatial memory, the novel object recognition, Y-maze test, nest building test, elevated plus maze test, open field test, tail suspension test and forced swimming test. The procedures followed were comparable to those described in earlier studies.

#### 2.3.1 Morris water maze test

The test was conducted in accordance with previously established protocols (Liu et al., 2020). The Morris water maze (MWM) test was performed in a circular tank measuring 150 cm in diameter and 35 cm in height (XR-XM101, Shanghai Xinruan Information Technology Co., Ltd.) in a dimly lit room. The water temperature was maintained between 22–25°C to prevent the mice from floating. The mice were placed in predefined pseudo-random locations. On day 0, the mice underwent four habituation training sessions. The platform was positioned 2 cm above the water’s surface, with the water left undyed. Mice were trained for five consecutive days, with each mouse undergoing four trials per day. A trial ended when the mouse located the platform or after 60 s had passed. A mouse was guided to the platform if it failed to locate the submerged platform within the time limit. All data were automatically recorded using a video tracking system (SuperMaze software, Shanghai Xinruan Information Technology Co., Ltd.).

#### 2.3.2 Y-maze spatial memory test

The test was conducted in accordance with previously established protocols (Dellu et al., 2000). The Y-maze consists of three identical arms (30 cm × 10 cm × 16 cm). During the training phase, one arm, referred to as the novel arm, was closed, while the remaining two arms were designated as the start arm and the familiar arm. Mice were introduced into the start arm and allowed to explore the start and familiar arms for 5 min. After 1 h, the mice were given 5 min to explore all three arms. The percentage of time spent in the novel arm was calculated using the formula: (time in the novel arm/total exploration time) × 100. The arena was cleaned with 70% ethanol to minimize olfactory cues before the commencement of trial for every mouse.

#### 2.3.3 Novel object recognition test

The novel object recognition (NOR) test comprises three stages. In the habituation stage, mice were allowed to explore an open field for 5 min. After 24 h, the training stage began, where mice explored the arena for 5 min with two identical objects placed in parallel. One hour later, during the testing stage, mice explored the arena again for 5 min, with one familiar object and one novel object placed side by side. To minimize olfactory cues, the open field was cleaned with 70% ethanol before each trial. Testing took place in a soundproof chamber maintained at 22–25°C. The arena was cleaned with 70% ethanol to minimize olfactory cues before the commencement of trial for every mouse. The novel object discrimination index (NODI) was calculated using the formula: (time spent with the novel object/total object exploration time) × 100 (Liu et al., 2022).

#### 2.3.4 Y-maze test

The Y-maze test, used to measure spatial working memory, was performed according to previously described methods (Kraeuter et al., 2019). After acclimatizing the mice, each arm of the Y-maze was marked with distinct visual cues. Mice were placed in the center of the maze and allowed to explore freely for 8 min. The total number of arm entries and spontaneous alternations were recorded. A spontaneous alternation was defined as consecutive entries into each of the three arms without repetition (e.g., arms one, two, three, or three, two, one, but not three, one, three). The arena was cleaned with 70% ethanol to minimize olfactory cues before the commencement of trial for every mouse. The alternation percentage was calculated using the formula: [number of spontaneous alternations/(total arm entries)] × 100.

#### 2.3.5 Nest building test

One hour before the onset of the dark phase, mice were individually housed in separate cages, each provided with a 3-gram pressed-cotton square. The next morning, the quality of the nest and the weight of the untorn nestlet were assessed. Nest scores were evaluated using a definitive 5-point rating scale based on a previously described system (Deacon, 2006).

#### 2.3.6 Elevated plus maze test

In the elevated plus maze test, mice were placed at the intersection of the four arms of the maze, and their behavior was recorded for 5 min. The arena was cleaned with 70% ethanol to minimize olfactory cues before the commencement of trial for every mouse. The primary behaviors observed include the time spent in and entries made into the open and closed arms. Activity in the open arms reflects a balance between the rodent’s preference for secure areas (e.g., closed arms) and their natural curiosity to explore new environments (Walf and Frye, 2007).

#### 2.3.7 Open field test

The open field test (OFT) was used to assess locomotor activity. The test was performed as described in previous studies. The apparatus consisted of a black metallic box measuring 60 × 80 × 50 cm, equipped with a video analysis system. Mice were placed in the center of the open field and allowed to explore freely for 6 min. After a 2-min adaptation period, the number of crossings was automatically recorded over the following 4 min (Lin et al., 2023). The arena was cleaned with 70% ethanol to minimize olfactory cues before the commencement of trial for every mouse.

#### 2.3.8 Forced swimming test

The forced swimming test (FST) was conducted in a cylindrical glass tank with a height of 25 cm, a diameter of 10 cm, and water maintained at 25°C. Each mouse was placed in the tank to adapt for 2 min, and then the cumulative immobility time was recorded for the following 4 min using ANY-maze software. Detailed procedures were based on previously published methods (Lin et al., 2023; Harro, 2018; Zhao et al., 2022).

#### 2.3.9 Tail suspension test

The tail suspension test (TST) was performed as previously described (Cryan et al., 2005). Mice were individually suspended upside down by the tail, using a clamp placed 1 cm from the tip. Each mouse was suspended for 6 min, and immobility time was recorded during the final 4 min when the mice remained completely motionless. The immobility time for each mouse during the last 4 min was analyzed using ANY-maze software.

### 2.4 Transmission electron microscope

After cardiac perfusion with saline and 4% paraformaldehyde-fixed solution, the CA1 region in the left side of hippocampus was sectioned and cut into 1 cm^3^ pieces and fixed in 2.5% glutaraldehyde at 4°C for 24 h. The sections were then washed three times in PBS, post-fixed with 1% osmium tetroxide, stained with 2% uranyl acetate, dehydrated in a graded series of ethanol and acetone, and embedded in epoxy resin. The sections were subsequently sliced into 70 nm thick sections using an ultramicrotome and stained with 4% uranyl acetate and 0.5% lead citrate after being mounted on copper grids. The ultrastructure of synapses in the hippocampus was observed using transmission electron microscopy (TEM) (FEI Company, Hillsboro, OR, United States), and synaptic morphometrics were analyzed. Synaptic parameters, including postsynaptic density (PSD), synaptic cleft width, synaptic interface curvature, and active zone length, were quantified using ImageJ software (version 1.53n; https://imagej.nih.gov/ij/).

### 2.5 Golgi-Cox staining

Golgi-Cox staining analyzed neuronal morphology Variations using the FD Rapid Golgi Stain Kit (Nanjing Well-Offex Biotechnology Co., Ltd., Nanjing, China; catalog number: PK401) as previously described (Du, 2019; Restivo et al., 2005). Briefly, the whole mouse brains were dissected, immersed in a mixture of solution A and solution B, and stored at room temperature in the dark for 14 days, with the solution replaced every 48 h. The brain tissues were then placed in solution C for 5 days, sectioned into 100-μm-thick slices using a vibratome, mounted on gelatin-coated slides, and stained with a mixture of solution D and solution E. The sections were dehydrated through a graded alcohol series, cleared with xylene, and covered with Permount. Images were captured using a digital camera attached to a microscope (Olympus Corp., Tokyo, Japan). Researchers blinded to the experimental conditions randomly selected dendritic shafts and spines of pyramidal neurons from CA1 region of the hippocampus for analysis. Morphological data, including total neurite length, individual neurite length, and neurite count per neuron, were analyzed using the NeuronJ plugin of ImageJ software. Spine density was estimated by counting the number of spines along a 10-μm section of a 30–50 μm-long distal dendritic branch using the Cell Counter plugin in ImageJ. Additionally, Sholl analysis was conducted to evaluate dendritic complexity with the Sholl plugin in ImageJ (Srinivasan et al., 2020). Images of Golgi-stained neurons were superimposed with concentric circles of increasing diameters (in 10-μm increments) around the soma (10–300 μm). The number of neurite intersections with each circle was counted manually, and the following indicators were calculated: (i) total intersections and (ii) maximum intersection distance.

### 2.6 Immunofluorescence

For image analysis of hippocampal immunofluorescence, sections were processed as previously described (Shi et al., 2021). Fresh brain tissues were soaked in a 4% paraformaldehyde-fixed solution and then transferred into a 30% sucrose solution for dehydration and cryoprotection. The mouse brain was embedded with an embedding agent and snap-frozen at −20°Cin a frozen sectioning machine, sectioned into 20-μm-thick slices, and washed three times for 10 min each. The sections were then treated with 1% H₂O₂ in PBS for 30 min. All sections were blocked with BSA (G5001, Servicebio) for 30 min and incubated overnight at 4°C with the indicated primary anti-Iba1^+^ antibody (Abcam, Cambridge, United Kingdom; catalog number Ab178847) and anti-beta-Amyloid-1-42 (HA721789, HUABIO, Hangzhou, China). Subsequently, the sections were incubated with a goat anti-rabbit IgG secondary antibody (GB23303, Servicebio Technology Co., Ltd., China) for 2 h at room temperature. Nuclei were stained using DAPI solution (G1012, Servicebio Technology Co., Ltd., China). Representative images were captured using a fluorescence microscope (OLYMPUS IX51), and the quantification of positively stained cells was performed using ImageJ.

### 2.7 RNA extraction and quantitative reverse transcription polymerase chain reaction

RNA extraction and quantitative reverse transcription polymerase chain reaction (RT-qPCR) were performed based on methods previously described (Yang et al., 2022). The fresh hippocampus and amygdala were collected and preserved at −80°C for RT-qPCR. The amygdala was removed from the brain where located in the most ventrocaudal part of the brain, near the hippocampus, in the frontal portion of the temporal lobe, below the subcortical nuclei, expanding to its basal structures (Nikolenko et al., 2020). Total RNA was extracted from hippocampal or amygdala tissues using TRIzol (Thermo Fisher Scientific, United States). The quantity of RNA was measured at 260 nm, and purity was assessed by the ratio of absorbance at 260 nm to 280 nm. Subsequently, 1 μg of purified RNA was reverse-transcribed into cDNA using the HiScript II Q RT SuperMix for qPCR (+gDNA wiper) (Vazyme Biotech Co., Ltd., Nanjing, China). qPCR was performed using the ChamQ SYBR qPCR Master Mix (Vazyme Biotech Co., Ltd., Nanjing, China) on a real-time PCR detection system (Roche, Switzerland). The relative mRNA expression levels were determined using the comparative CT method (2^−ΔΔCt^) and normalized to the expression of the housekeeping gene β-actin. Primer sequences were shown in Table 1.

**Table 1:** The RT-qPCR primer sequences used in this study.

No.	Gene symbol	Forward primer (5′–3′)	Reverse primer (5′–3′)
1	TNF-α	CTTGTTGCCTCCTCTTTTGCTTA	CTTTATTTCTCTCAATGACCCGTAG
2	IL-6	CTGCTCATTCACGAAAAGGGA	TCACAGAAGGAGTGGCTAAGGACC
3	IL-1β	TGGGAAACAACAGTGGTCAGG	CTGCTCATTCACGAAAAGGGA
4	β-actin	CGTGGGCCGCCCTAGGCACCA	TTGGCCTTAGGGTTCAGGGGGG

### 2.8 Western blotting

Western blot assays were performed as described previously (Wu et al., 2021). The hippocampus was collected and preserved at −80°C for western blotting. Total protein concentration from hippocampal samples was determined using a BCA protein assay kit. Equal amounts of protein were separated via sodium dodecyl sulfate-polyacrylamide gel electrophoresis (SDS-PAGE) and transferred to polyvinylidene difluoride (PVDF) membranes. The membranes were blocked with 5% non-fat milk for 1 h at room temperature, followed by overnight incubation at 4°C with primary antibodies. Primary antibodies included anti-BDNF (Alomone Labs, Jerusalem, Israel; catalog number ANT-010), anti-PSD95 (Cell Signaling Technology; catalog number 3450) and β-actin (ABclonal, AS003). After washing the membranes three times in TBST, they were incubated with an HRP-linked anti-rabbit IgG secondary antibody (ABclonal, AS014) for 1 h at room temperature. After three additional washes, protein bands were detected using Clarity^™^ ECL Western Blot Substrate (Bio-Rad, 1705060) and visualized with the ChemiDoc Touch imaging system (Bio-Rad). Protein expression levels were normalized to β-actin expression. The procedure for image quantification of western blot band involves scanning the blot to obtain a digital image, followed by ImageJ software analysis. First, background correction is applied to remove non-specific signals. Then, the intensity of each protein band is measured by defining areas of interest. The intensity values are normalized to β-actin, to account for variations in protein loading. Finally, results are analyzed and quantified relative to control samples or other experimental conditions.

### 2.9 Statistical analysis

Data were presented as mean ± standard error of the mean (SEM) and analyzed using GraphPad Prism software 8.0. One-way analysis of variance (ANOVA) was used to compare four groups, followed by the *post hoc* Tukey–Kramer test for multiple comparisons. *p*-value <0.05 was considered to indicate statistical significance.

## 3 Results

### 3.1 Diethyl butylmalonate ameliorates cognitive deficits in 5×FAD mice

**Figure 1:**
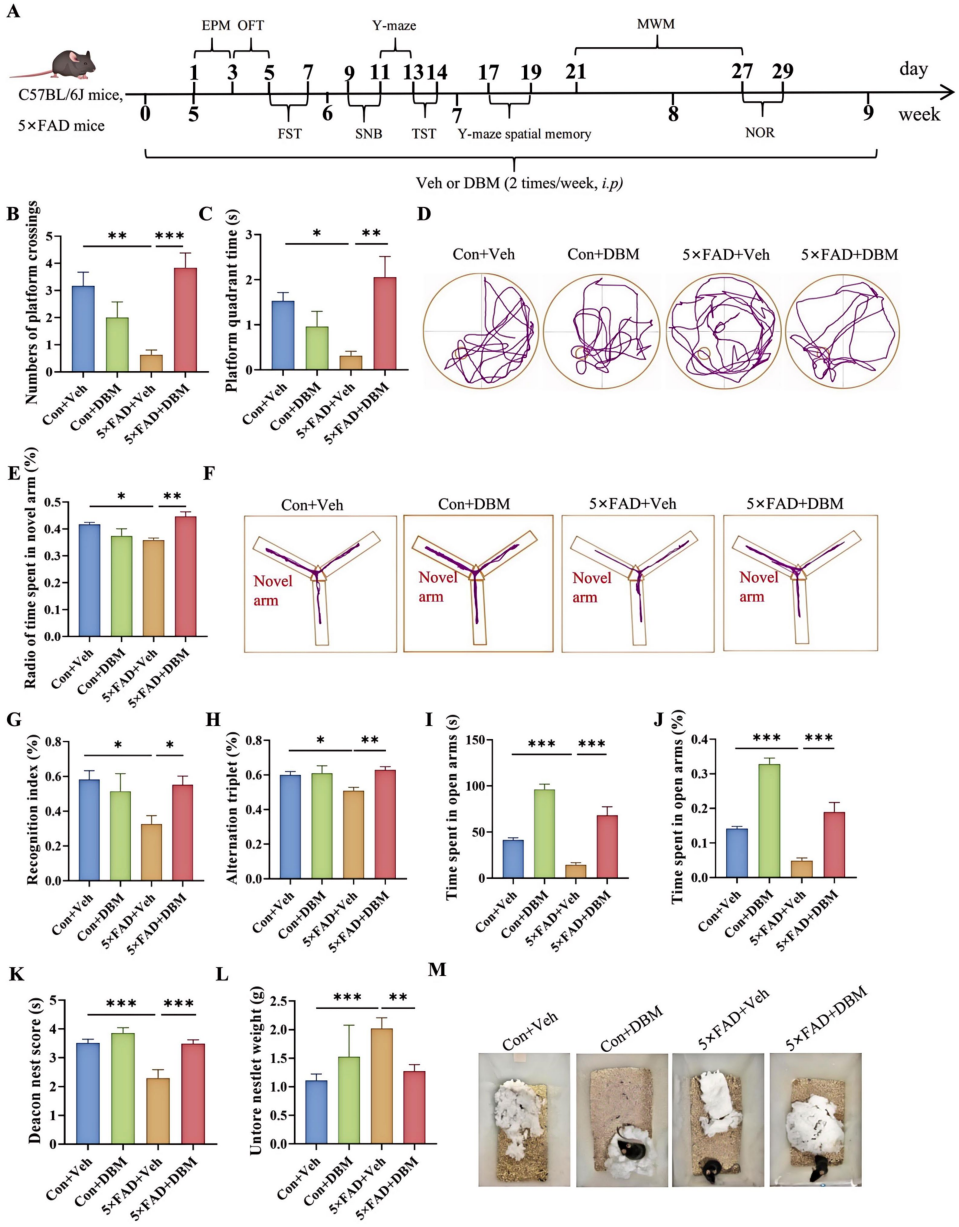
Diethyl butylmalonate ameliorates cognitive deficits in 5×FAD mice. **(A)** Schematic timeline for DBM treatment on cognitive deficits and depression in 5×FAD mice. **(B)** Number of platform crossings and **(C)** the platform quadrant time in the Morris water maze were recorded. **(D)** Representative track plots of Con + Veh, Con + DBM, 5×FAD + Veh and 5×FAD + DBM groups recorded by SMART video tracking system in the testing place. **(E)** Radio of time spent in novel arm was recorded in Y-maze spatial memory test. **(F)** Representative track plots of Con + Veh, Con + DBM, 5×FAD + Veh and 5×FAD + DBM groups recorded by SMART video tracking system in the testing place. **(G)** Recoginiton index and **(H)** total time were recorded in novel object recognition test. **(I)** Time spent in open arms and **(J)** the index of time spent in open arms were recorded in Y-maze test. **(K)** Deacon nest score and **(L)** untorn nestlet weight (amount of untorn nesting material). **(M)** Representative images of nesting result in Con + Veh, Con + DBM, 5×FAD + Veh and 5×FAD + DBM groups. *n* = 12 mice for each group. Values are mean ± SEM. ^*^p < 0.05, ^**^p < 0.01, and ^***^p < 0.001.

The cognitive function (including recognition memory, spatial memory and ability to perform activities of daily living) can be evaluated by Morris water maze, Y-maze spatial memory, NOR, Y-maze test and nest building test (Hattiangady et al., 2014; Broadbent et al., 2004; Dellu et al., 2000; Deacon, 2006; Yoshizaki et al., 2020). The strategy was shown in Figure 1A. In brief, DBM administration (twice per week), started from the beginning until the ending of behavioral tests. In Morris water maze test, the numbers of platform crossings and the platform quadrant time were decreasing in 5×FAD mice compared with the Con + Veh mice (*p* < 0.01, Figure 1B; *p* < 0.05, Figures 1C,D); however, DBM supplementation significantly improved exploration ability, increasing the numbers of platform crossings and the platform quadrant time in 5×FAD mice (*p* < 0.001, Figure 1B; *p* < 0.01, 1B; *p* < 0.05, Figures 1C,D). In Y-maze spatial memory test, DBM supplementation improved spatial recognition memory impairment in 5×FAD mice, as demonstrated by an increase in the ratio of time spent in the novel arm (percentage of time spent in novel arm) (both *p* < 0.01, Figures 1E,F). In NOR, DBM supplementation could prevent recognition memory impairment in 5×FAD mice, with an increase in the novel object recognition index (percentage of time spent with the novel object) (*p* < 0.05, Figure 1G), and there is no significant difference in the total exploration time (*p* > 0.05, Figure 1H). In the Y-maze test, the time spent in open arms and the index of it in the 5×FAD mice was clearly lower than that in the Con + Veh mice, while DBM significantly improved the exploration ability in 5×FAD mice (*p* < 0.001, Figures 1I,J). In NB, the nesting ability of 5×FAD mice was reduced compared to the control + Veh mice and DBM supplementation could recover the nesting building ability (*p* < 0.001, Figure 1K). In contrast, the untore nestlet weight (nest-building deficit) of 5×FAD + DBM mice was significantly decreased compared with that of the 5×FAD group (*p* < 0.01, Figures 1L,M). Overall, these findings indicate that DBM ameliorates cognitive impairment.

### 3.2 Diethyl butylmalonate attenuates depression in 5×FAD mice

**Figure 2:**
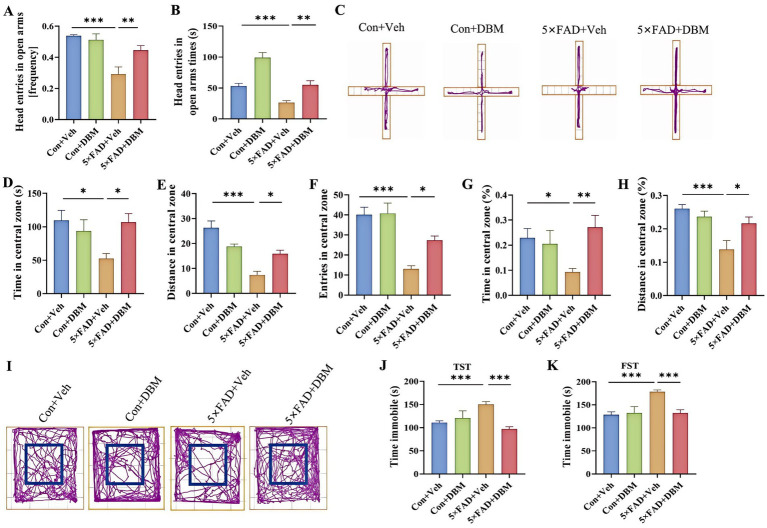
Diethyl butylmalonate improves depression in 5×FAD mice. **(A)** The frequency entries in open arms, **(B)** head entries in open arms times, **(C)** representative track plots of Con + Veh, Con + DBM, 5×FAD + Veh and 5×FAD + DBM groups recorded by SMART video tracking system in the elevated plus maze test. **(D)** Time in the central zone, **(E)** distance in the central zone, **(F)** entries in the central zone, **(G)** index of time in the central zone and **(H)** index of distance in the central zone were recorded in the open field test. **(I)** Representative track plots of Con + Veh, Con + DBM, 5×FAD + Veh and 5×FAD + DBM groups recorded by SMART video tracking system in the open field test. **(J)** Time immobile was recorded in tail suspension test. **(K)** Time immobile in forced swimming test. *n* = 12 mice for each group. Values are mean ± SEM. ^*^p < 0.05, ^**^p < 0.01, and ^***^p < 0.001.

We next evaluated the effects of DBM on depression-like behaviors in 5×FAD mice using elevated plus maze test, open field test (OFT), tail suspension test (TST) and forced swimming test (FST) (Yoshizaki et al., 2020; Dang et al., 2022; Sithisarn et al., 2013). In the elevated plus maze test, 5×FAD mice showed a significantly reduced frequency of head entries in open arms and the time of head entries in open arms as compared to the Con + Veh mice (*p* < 0.001, Figures 2A–C); while DBM supplementation significantly increased the novel object discrimination index (*p* < 0.01, Figures 2A–C). In OFT, DBM supplementation induced a protective effect on 5×FAD mice represented by an increase in the time in the central zone, the entries in the central zone, the entries in the central zone, the index of the time in the central zone and the index of the distance in the central zone (all *p* < 0.05, Figures 2D–I). Subsequently, in TST, we found that 5×FAD mice displayed a higher level of immobility in comparison to the Con + Veh mice, while DBM supplementation could attenuate the depression-like behavior in 5×FAD mice (*p* < 0.001, Figure 2J). In FST, the result of the time immobile was the same as TST (*p* < 0.001, Figure 2K). Overall, these results suggest that DBM improves depression in 5×FAD mice.

### 3.3 Diethyl butylmalonate mitigated neurite impairment in the hippocampus of 5×FAD mice

**Figure 3:**
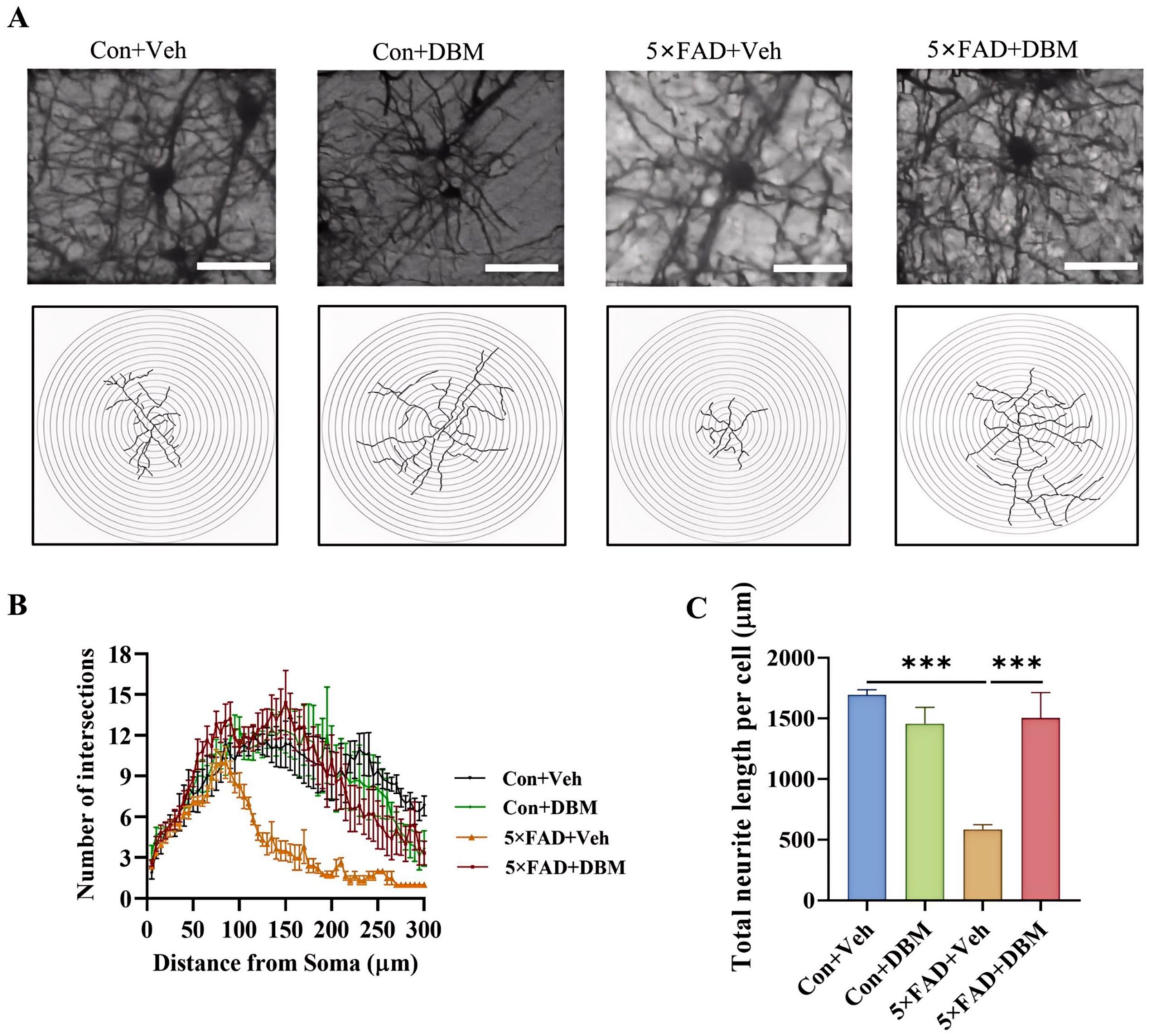
Diethyl butylmalonate relieves neuron degeneration in the hippocampus of female 5×FAD mice. Golgi-Cox staining was used to analyze the neuronal morphological variations in the cornu ammonis 1 (CA1) region of the hippocampus of female mice. **(A)** Representative images of pyramidal neurons. **(B)** Sholl analysis of dendritic branching complexity of pyramidal neurons. **(C)** Total neurite length per cell. *n* = 3, scale bars: 100 μm. Values are mean ± SEM. ^*^p < 0.05, ^**^p < 0.01, and ^***^p < 0.001.

The hippocampus, lying just beneath the neocortex, is implicated in cognitive processing, social recognition and memory (Alexander et al., 2016; Zhou et al., 2015). Neurite impairment in the hippocampus is the key event in AD (Wong et al., 2002). Using Golgi-Cox staining, we investigated the effects of DBM supplementation on neurite impairment in the hippocampus, a critical brain region responsible for cognition or memory. Sholl analysis showed that 5×FAD + DBM mice had increased dendritic complexity by increasing the sum number of dendritic intersections and the distance of the maximum intersections from the soma (Figures 3A,B). A significant decrease in the total neurite length per cell was observed in 5×FAD mice, while DBM supplementation increased the length of total neurite per cell (*p* < 0.001, Figures 3A,C). These results indicate that DBM can prevent neurite degeneration and increase neuronal complexity in the hippocampus of 5×FAD mice.

### 3.4 Diethyl butylmalonate prevents synaptic ultrastructural impairment in the hippocampus of 5×FAD mice

**Figure 4:**
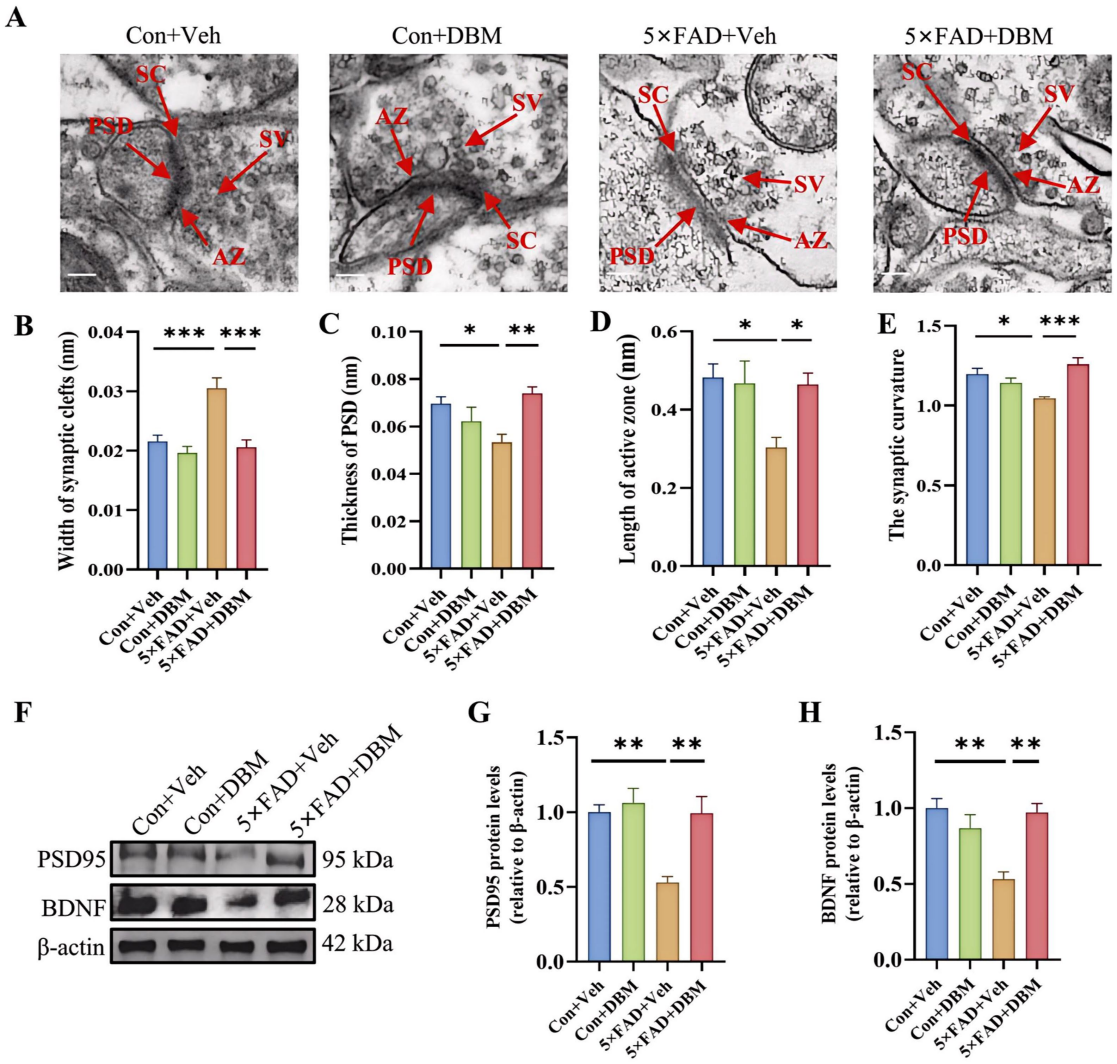
Diethyl butylmalonate ameliorates synaptic ultrastructural impairment in the hippocampus of 5×FAD female mice. **(A–E)** Synaptic ultrastructure in the cornu ammonis 1 (CA1) region of the hippocampus of mice was analyzed by transmission electron microscope (*n* = 3, scale bars: 100 nm): **(A)** representative images of synaptic ultrastructure, **(B–E)** the statistical analysis of the synaptic ultrastructure associated indexes (*n* = 3, 5 figures for each group): **(B)** width of synaptic cleft, **(C)** thickness of PSD, **(D)** length of active zone, **(E)** synaptic curvature. **(F–H)** The protein expression of PSD95 **(F, G)**, BDNF **(F, H)** in the hippocampus of mice (*n* = 4). Values are mean ± SEM. ^*^p < 0.05, ^**^p < 0.01, and ^***^p < 0.001.

Impairment of synaptic ultrastructure has been implicated in cognitive impairment and AD (Head et al., 2009; Whitfield et al., 2014). Using transmission electron microscopy, we observed an increased width of the synaptic cleft (*p* < 0.001, Figures 4A,B) and a reduction in PSD thickness (*p* < 0.05, Figures 4A,C) in hippocampal CA1 region of 5×FAD mice. However, DBM supplementation shortened the width of synaptic cleft and prevented the decrease in the thickness of PSD (*p* < 0.001, Figure 4B; *p* < 0.01, Figure 4C). It also increase the length of active zone and the synaptic curvature (*p* < 0.001, Figure 4E; *p* < 0.05, Figure 4D) in 5×FAD mice. Furthermore, we found that DBM supplementation prevented the reduction of postsynaptic density protein 95 (PSD95) and brain-derived neurotrophic factor (BDNF) in the hippocampus of the 5×FAD mice (*p* < 0.01, Figures 4F–H). However, DBM did not obviously reduce Aβ deposition in the hippocampus of 5×FAD mice (Supplementary Figure S1). Therefore, DBM plays the protective effect on cognitive memory via improvement of synaptic ultrastructure damage in 5×FAD mice.

### 3.5 Diethyl butylmalonate ameliorates neuroinflammation in the hippocampus of 5×FAD mice

**Figure 5:**
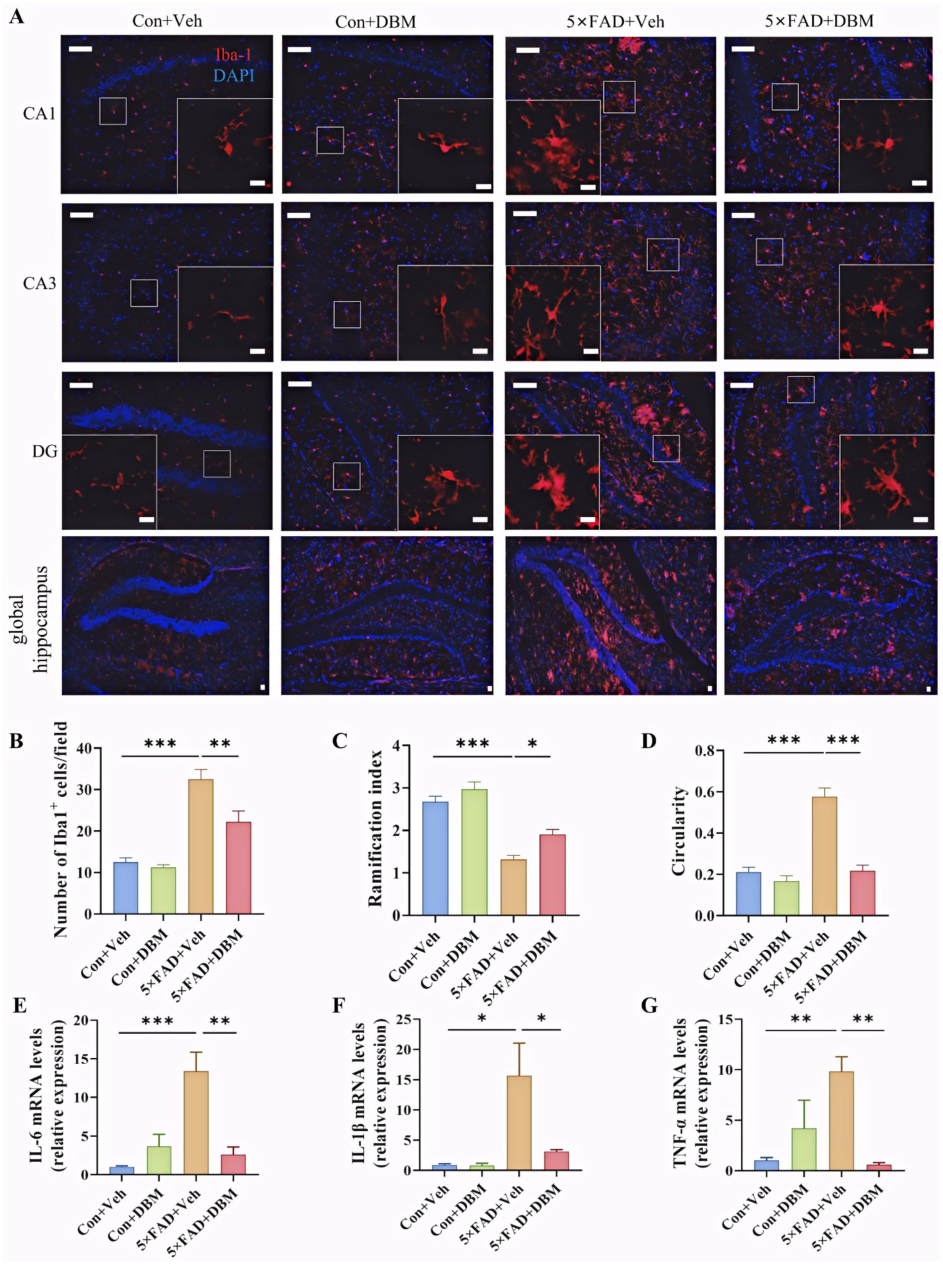
Diethyl butylmalonate ameliorates the neuroinflammation in the hippocampus of 5×FAD female mice. **(A)** Representative immunofluorescent staining of the Iba-1^+^ cells in the CA1, CA3 and DG regions of hippocampus and the global hippocampus (*n* = 3). **(B)** The quantification of Iba-1 cells number in the hippocampus (*n* = 3, 10 images per mouse per region, scale bar: 50 μm). **(C)** The ramification of microglia in the hippocampus (*n* = 3, 10 images per mouse per region, scale bar: 50 μm). **(D)** The circularity of microglia in the hippocampus (*n* = 3, 10 images per mouse per region, scale bar: 50 μm). **(E–G)** The mRNA expression of IL-1β, IL-6 and TNF-α in the hippocampus (*n* = 4). Con + Veh, control mice with vehicle control treatment; Con + DBM, control mice with DBM treatment; 5×FAD + Veh, 5×FAD mice with vehicle control treatment; 5×FAD + DBM: 5×FAD mice with DBM treatment. Values are mean ± SEM. ^*^p < 0.05, ^**^p < 0.01, and ^***^p < 0.001.

Activation of microglia is implicated in neuroinflammation and is considered critical in the pathogenesis of AD (Leng and Edison, 2021; Subhramanyam et al., 2019; Thakur et al., 2023; Cunningham, 2013). Here, the morphological alteration of microglia was investigated by immunofluorescent staining with Iba1^+^ (microglia marker) antibody (Figure 5A). We observed the increased microglia number in the hippocampus of the 5×FAD group (*p* < 0.001, Figure 5B). In the 5×FAD group, the majority of Iba1^+^ microglia showed the morphology of activated microglia with elongated soma and fewer branches in the hippocampus of the hippocampus (*p* < 0.001, Figures 5C,D). In the control and DBM supplementation groups, the Iba1^+^ microglia showed the characteristic of resting microglia consisting of a rod-shaped cell body with thin processes (*p* < 0.05, Figure 5A; *p* < 0.001, Figure 5D). These results suggest that DBM inhibits the activation of microglia.

Furthermore, we investigated whether DBM could improve the neuroinflammation in the hippocampus. DBM supplementation significantly prevented an increase in the microglia number in these areas (*p* < 0.01, Figure 5B) and the circularity in 5×FAD mice (*p* < 0.001, Figure 5D). DBM supplementation also prevented a decrease in the ramification index in 5×FAD mice (*p* < 0.05, Figure 5C), with a decrease in the mRNA levels of pro-inflammatory cytokines (IL-1β, IL-6, TNF-α) in 5×FAD mice (*p* < 0.05, Figures 5E,G). These results showed that DBM attenuates the neuroinflammation in the hippocampus of 5×FAD mice.

### 3.6 Diethyl butylmalonate reduces neuroinflammation in the amygdala of 5×FAD mice

**Figure 6:**
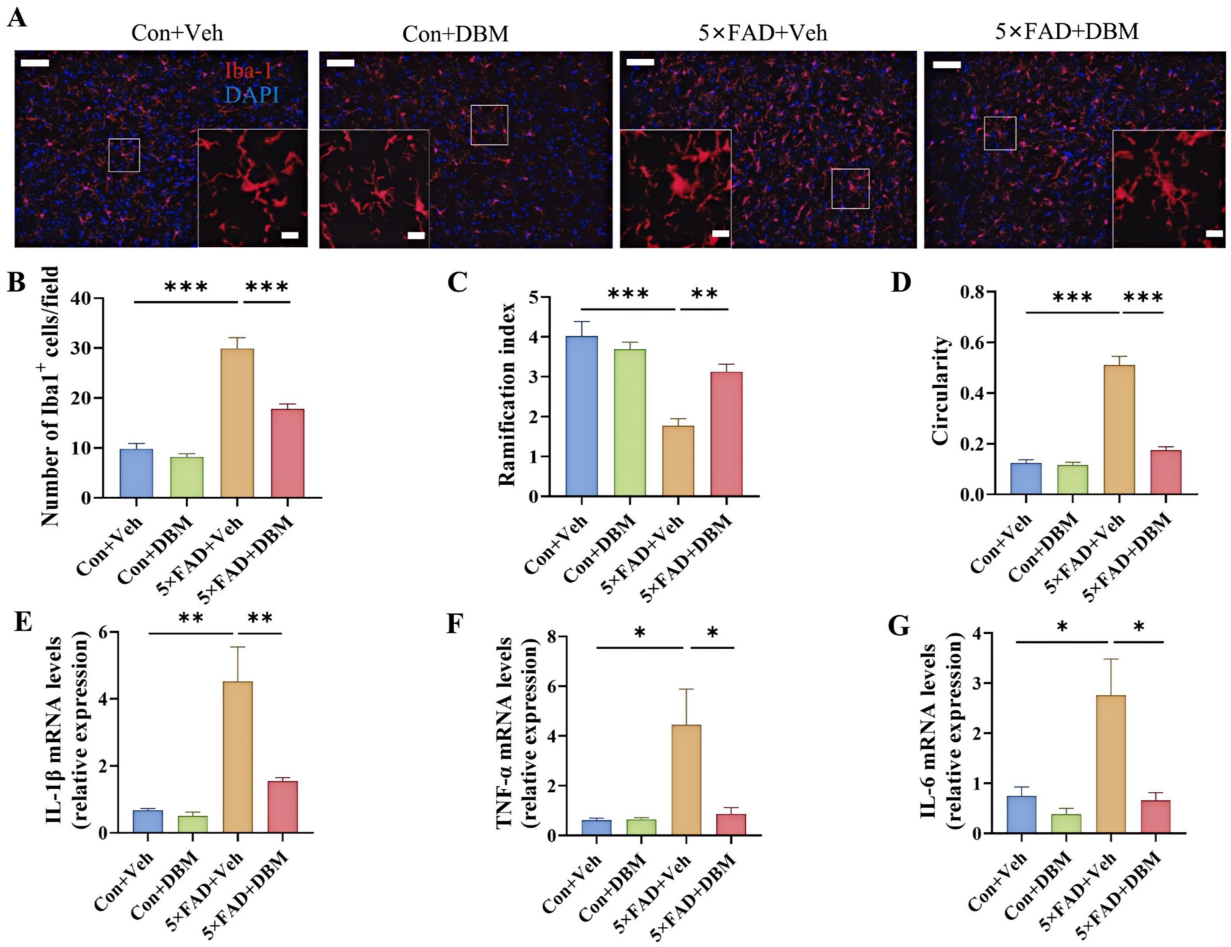
Diethyl butylmalonate ameliorates the neuroinflammation in the amygdala of 5×FAD female mice. **(A)** Representative immunofluorescent staining of the Iba-1 cells in the amygdala (*n* = 3). **(B)** The quantification of Iba-1 cells number in the amygdala (*n* = 3, 10 images per mouse per region, scale bar: 50 μm). **(C)** The ramification of microglia in the amygdala (*n* = 3, 10 images per mouse per region, scale bar: 50 μm). **(D)** The circularity of microglia in the amygdala (*n* = 3, 10 images per mouse per region, scale bar: 50 μm). **(E–G)** The mRNA expression of IL-1β, IL-6 and TNF-α the amygdala (*n* = 4). Con + Veh, control mice with vehicle control treatment; Con + DBM, control mice with DBM treatment; 5×FAD + Veh, 5×FAD mice with vehicle control treatment; 5×FAD + DBM: 5×FAD mice with DBM treatment. Values are mean ± SEM. ^*^p < 0.05, ^**^p < 0.01, and ^***^p < 0.001.

Neuroinflammation in the amygdala is closely associated with depression (Zheng et al., 2021). We further characterized the effects of DBM on neuroinflammatory profiles in the amygdala. We observed the increased microglia number in the amygdala of the 5×FAD group and DBM supplementation significantly reduced the microglia number in this region (*p* < 0.001, Figures 6A,B). Correspondingly, in compared with Con + Veh and 5×FAD + DBM groups, the decreased ramification index and increased circularity of microglia were observed in 5×FAD group (*p* < 0.01, Figure 6C; *p* < 0.001, Figure 6D). Furthermore, DBM supplementation significantly prevented the upregulation of IL-1β, IL-6 and TNF-α mRNA expression in the amygdala of 5×FAD (*p* < 0.01, Figure 6E; *p* < 0.05, Figures 6F,G). These results show that DBM attenuates the neuroinflammation in the amygdala of 5×FAD mice.

## 4 Discussion

**Figure 7:**
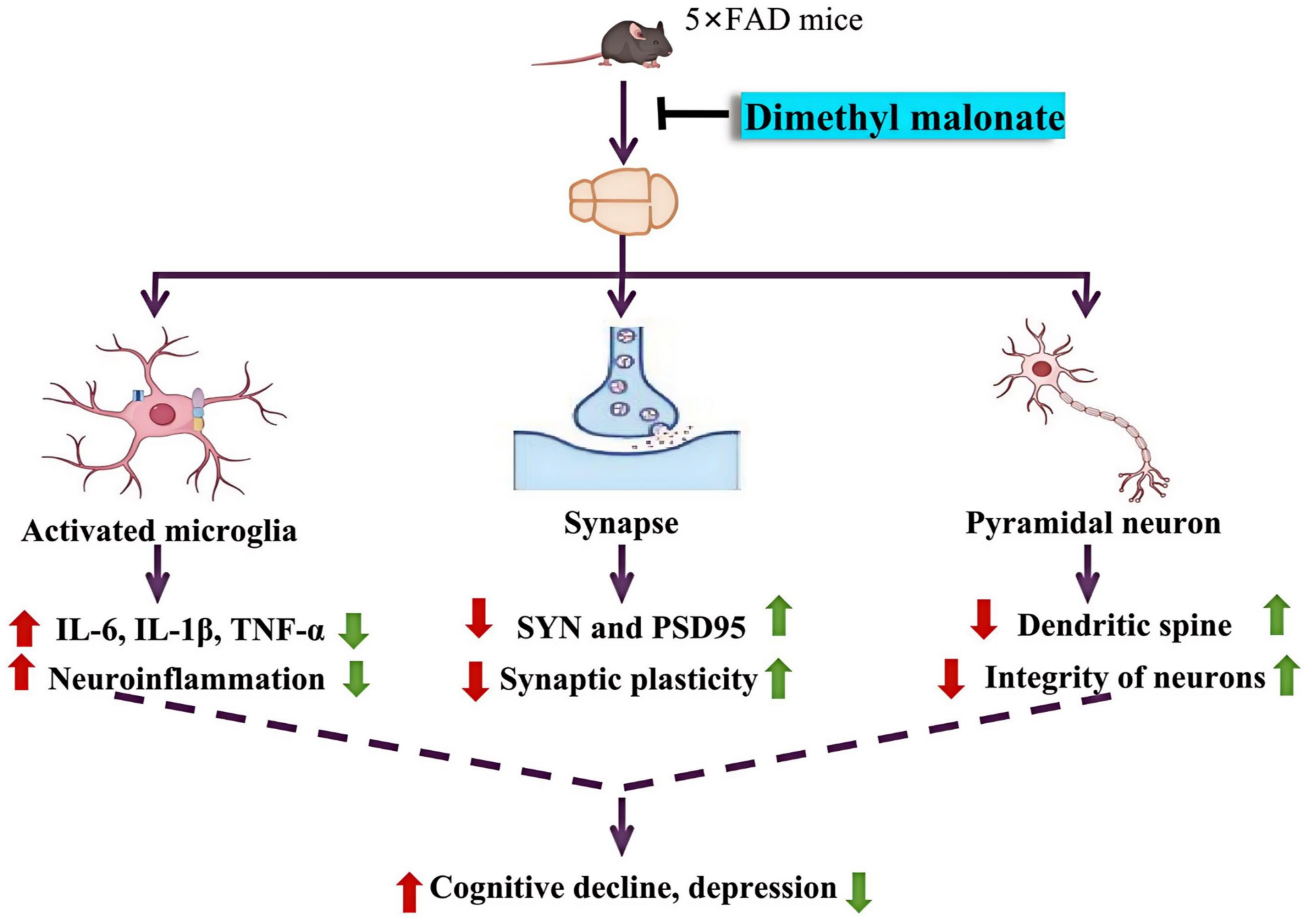
Graphical summary of DBM’s protective effects on cognitive impairment and depression in 5×FAD mice. In 5×FAD mice, activation of microglia induces the production of pro-inflammatory cytokines in the brains, including the hippocampus and amygdala, reduces the integrity of pyramidal neurons, and impairs synaptic ultrastructure along with the deficits of synapse-related proteins, and consequently causing cognitive decline and depression. However, DBM supplementation alleviates these pathological alterations by inhibiting microglia activation and neuroinflammation. Red arrows represent key events in 5×FAD mice; green arrows represent the amelioration by DBM administration.

The discovery of new effective drugs for AD is imperative yet challenging (Cummings et al., 2023; Price and Duman, 2019). Here, we investigated the effects of DBM on abnormal behaviors in 5×FAD mice. We showed that DBM supplementation alleviates cognitive impairment and depression along with the improvement of neurite outgrowth, synaptic ultrastructure damage and neuroinflammation (Figure 7). Our findings support that DBM may be a drug candidate for treating neurodegeneration.

AD is a progressive form of dementia marked by cognitive and memory deficits (Leng and Edison, 2021; Fang et al., 2021). Also, depression is one of the most common neuropsychiatric disorders that accompanies AD, appearing in up to 50% of patients (Chi et al., 2014). Neuroinflammation is one of the hallmarks of neuropsychiatric disorders (Leng and Edison, 2021). It is reported that activated microglia release TNF-α, IL-1β and IL-6, and other pro-inflammatory factors that mediate secondary brain damage (Heneka et al., 2015). These pro-inflammatory factors cause harm and neuron loss, ultimately leading to learning and memory dysfunction (Zhao et al., 2021). In addition, neuroinflammation can also adversely affect cognitive function by interfering with normal signaling between neurons (Cunningham, 2013). Thus, neuroinflammation mediated by microglia activation is key for AD progression. Here, using classic behavioral tests, we showed that DBM, a competitive inhibitor of SDH, can attenuate cognitive deficits and depression in 5×FAD mice, which is associated with reduced neuroinflammation.

It has been reported that SDH increases mitochondrial succinate oxidation and mitochondrial membrane potential, thereby promoting ROS production. Inhibiting SDH in macrophages prevents the induction of a range of pro-inflammatory factors typified by IL-1β (Mills et al., 2016). Here, we showed that *in vivo* DBM administration reduces the accumulation of microglia and inhibits the upregulation of pro-inflammatory cytokines in the hippocampus and amygdala, two brain regions associated with cognitive deficits and depression. It is also reported that DBM can downregulate pro-inflammatory response in LPS-stimulated microglia cells (Zhao et al., 2019). Therefore, DBM’s anti-inflammatory effect may be attributed to pro-cognition and anti-depression function in AD mice. Furthermore, microglia are able to bind to soluble β-amyloid (Aβ) oligomers and Aβ fibrils via cell-surface receptors and Toll-like receptors in AD (Thakur et al., 2023; Heneka et al., 2015). The deposition in β-amyloid (Aβ) protein are characteristic of the AD brain (Ghosh et al., 2015; Holtzman et al., 2011). However, we only observed a slight decrease of Aβ deposition in the hippocampal region of 5×FAD mice after DBM treatment.

Neurite outgrowth and synaptic plasticity are manipulated by neuroinflammation, and consequently, cognitive deficits and depression (Cai et al., 2019; Heneka et al., 2015). We observed that DBM administration increases the structural integrity and the complexity of neurons in the hippocampus. Furthermore, the dysregulation of synaptic ultrastructure has been implicated in AD patients (Head et al., 2009; Whitfield et al., 2014). Here, we found that the impairment of synaptic ultrastructure in the hippocampus of AD mice is attenuated by DBM treatment. Previous studies have shown the deficits of postsynaptic proteins PSD95 and synaptic plasticity-related protein BDNF in synaptic plasticity, cognitive function and depression (Choo et al., 2017; Wang et al., 2024; Liu et al., 2010). This study showed that DBM supplementation alleviates the protein deficits of PSD95 and BDNF. These data further explain why DBM improves cognitive deficits and depression.

Overall, our study shows that DBM has a beneficial effect on cognitive deficits and depression in AD mice via manipulating neuroinflammation, which provides a novel drug candidate for treating neurodegenerative diseases, including AD. However, this study also has several shortcomings. We only evaluated the protective effect of DBM in female AD mice, since that female mice display accelerated disease progression (Poon et al., 2023). It should be pointed out that DBM’s effect on male AD mice should also be investigated in the future. Moreover, we did a series of behavioral tests to confirm the effect of DBM on cognitive impairment and depression. However, in Morris Water Maze test, we did not record the learning curve (the latency time to get on the platform), which might affect the behavioral performance.

## Data Availability

The original contributions presented in the study are included in the article/Supplementary material, further inquiries can be directed to the corresponding authors.
