# Supplementary material for: Diethyl butylmalonate attenuates cognitive deficits and depression in 5×FAD mice
Source: Front Neurosci. 2024 Nov 11;18:1480000. doi: 10.3389/fnins.2024.1480000 (PMC11586351; doi:10.3389/fnins.2024.1480000)
Supplement: Supplementary file 1 [file Data_Sheet_1.DOCX]

**Diethyl butylmalonate attenuates cognitive deficits and depression**

**in 5×FAD mice**

**Lai Yuan^1,2†^, Ge Song^1,2†^, Wangwei Xu^3,4†^, Shuni Liu^1,2†^, Yongsheng Zhang^1,2^, Wei Pan^1^, Xiaohui Ding^1^, Linlin Fu^1*^, Qisi Lin^3*^, Fenfen Sun^1*^**

^1^ Jiangsu Key Laboratory of Immunity and Metabolism, Department of Pathogen Biology and Immunology, Xuzhou Medical University, Xuzhou, Jiangsu, China

^2^ The First Clinical Medical College, Xuzhou Medical University, Xuzhou, Jiangsu, China

^3^ Jiangsu Key Laboratory of New Drug Research and Clinical Pharmacy, Xuzhou Medical University, Xuzhou, Jiangsu, China

^4^ Suqian Affiliated Hospital of Xuzhou Medical University, Suqian, Jiangsu, China

**^†^**These authors share the first authorship of this work.

^*^**Correspondence**:

fen_1208@163.com (Fenfen Sun); qslin074@126.com (Qisi Lin); 363504347@qq.com (Linlin Fu)


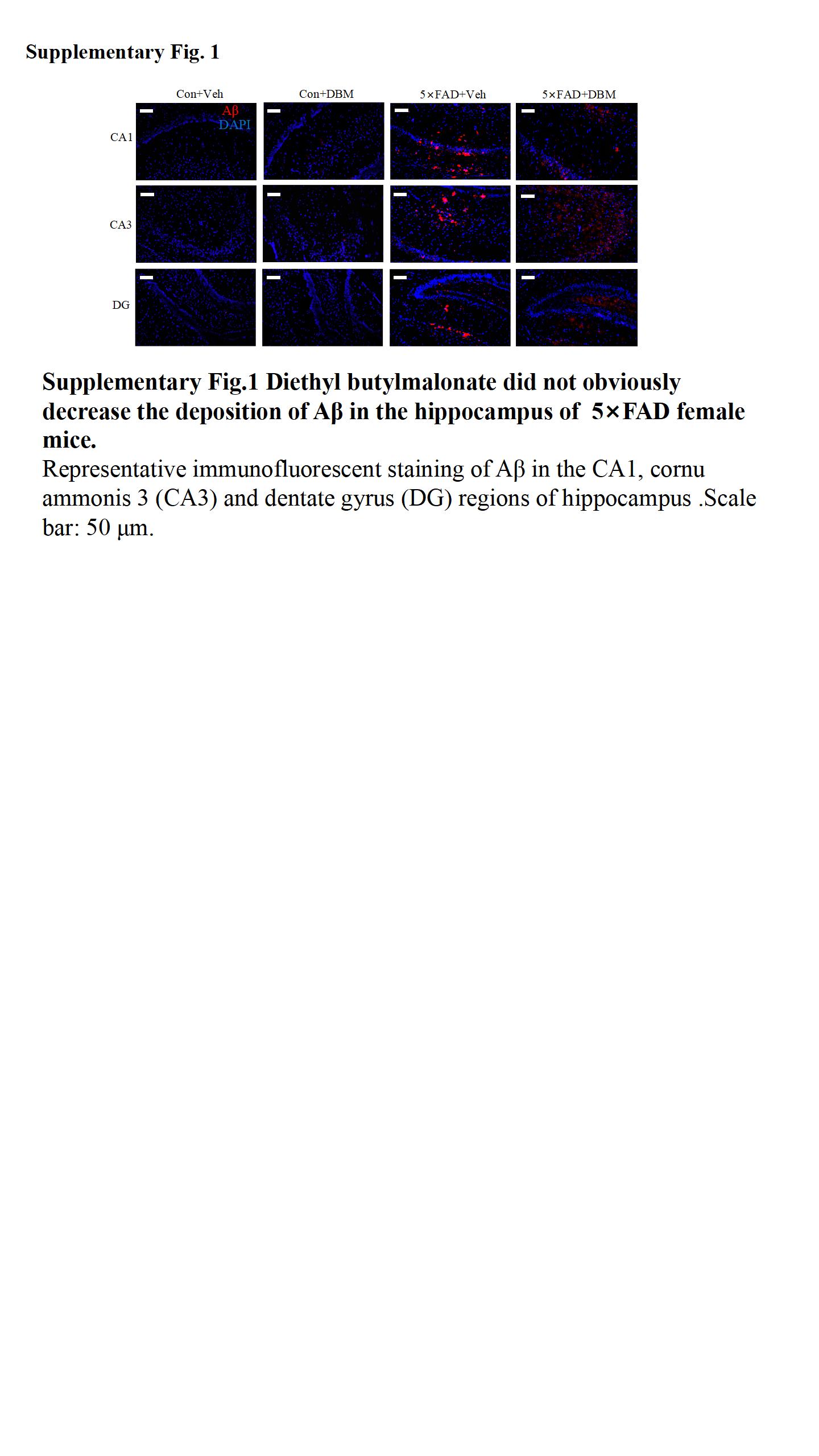
**Supplementary Fig.1 Diethyl butylmalonate did not obviously decrease the deposition of Aβ in the hippocampus of 5×FAD female mice.**

Representative immunofluorescent staining of Aβ in the cornu ammonis (CA1), cornu ammonis 3 (CA3) and dentate gyrus (DG) regions of hippocampus. Scale bar: 50 μm.
